# Supplementary material for: The novel ECM protein SNED1 mediates cell adhesion via the RGD-binding integrins α5β1 and αvβ3
Source: J Cell Sci. 2025 Jan 22;138(2):JCS263479. doi: 10.1242/jcs.263479 (PMC11828466; doi:10.1242/jcs.263479)
Supplement: Supplementary information [file joces-138-263479-s1.pdf]

J. Cell Sci.: doi:10.1242/jcs.263479: Supplementary information

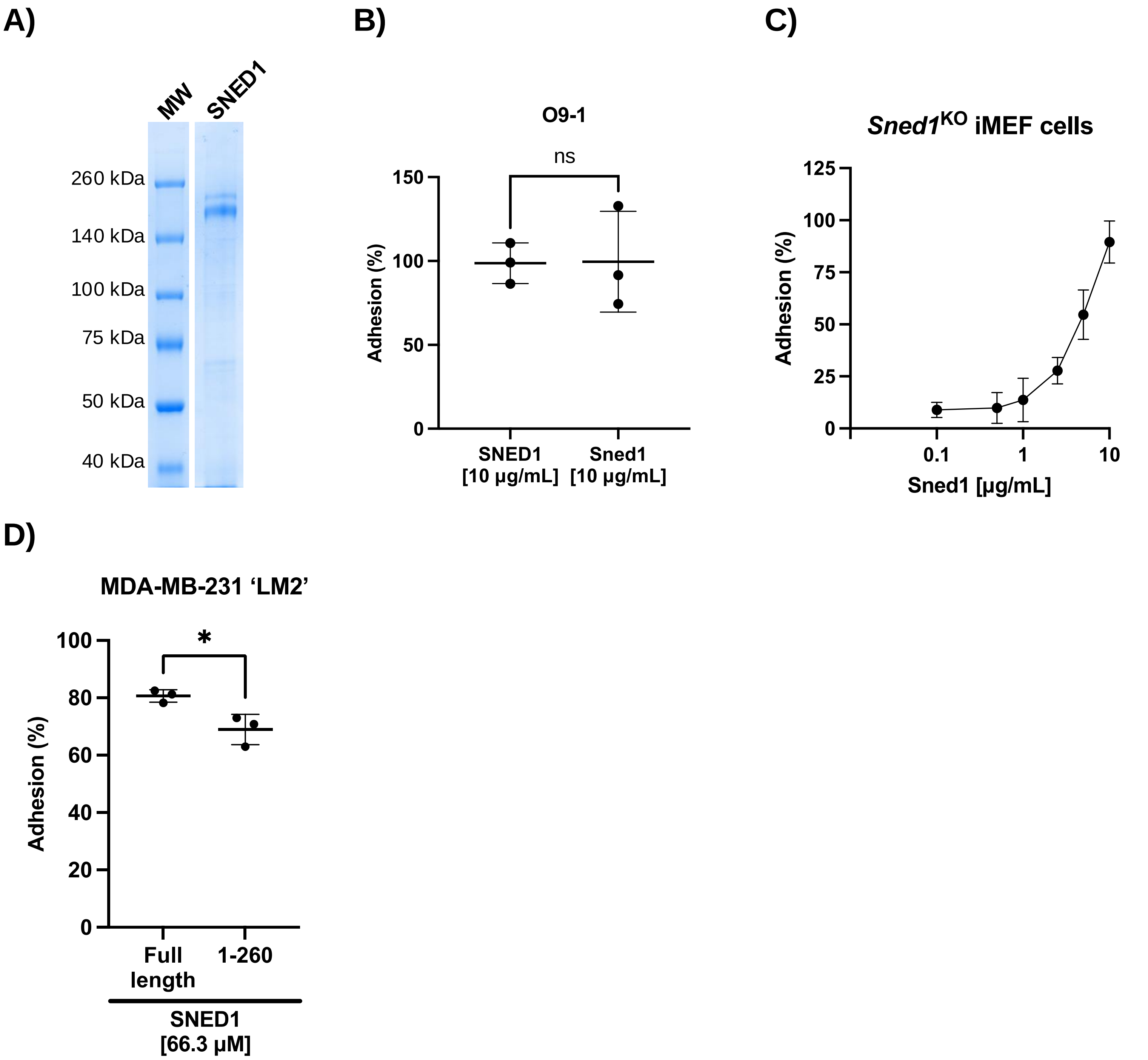

Fig. S1. Cell adhesion on human SNED1, murine Sned1 and SNED1$^{1\text{-}260}$

A) Coomassie-blue-stained polyacrylamide gel showing the quality and purity of affinity-purified full-length SNED1-His.

B) Murine O9-1 neural crest cells adhere to the same extent to human SNED1 and murine Sned1. Data is represented as mean ± SD from three biological replicates. Unpaired Student's two-tailed t-test with Welch's correction was performed to test statistical significance. ns: non-significant.

C) Graph showing the adhesion of immortalized embryonic fibroblast cells isolated from *Sned1* knockout mice (*Sned1*^{KO} iMEF) on increasing concentrations of Sned1.

D) Graph showing MDA-MB-231 'LM2' breast cancer cell adhesion on 66.3 µM of full length SNED1 or SNED1$^{1\text{-}260}$. Unpaired Student's two-tailed t-test with Welch's correction was performed to test statistical significance. *P<0.05

Journal of Cell Science • Supplementary information

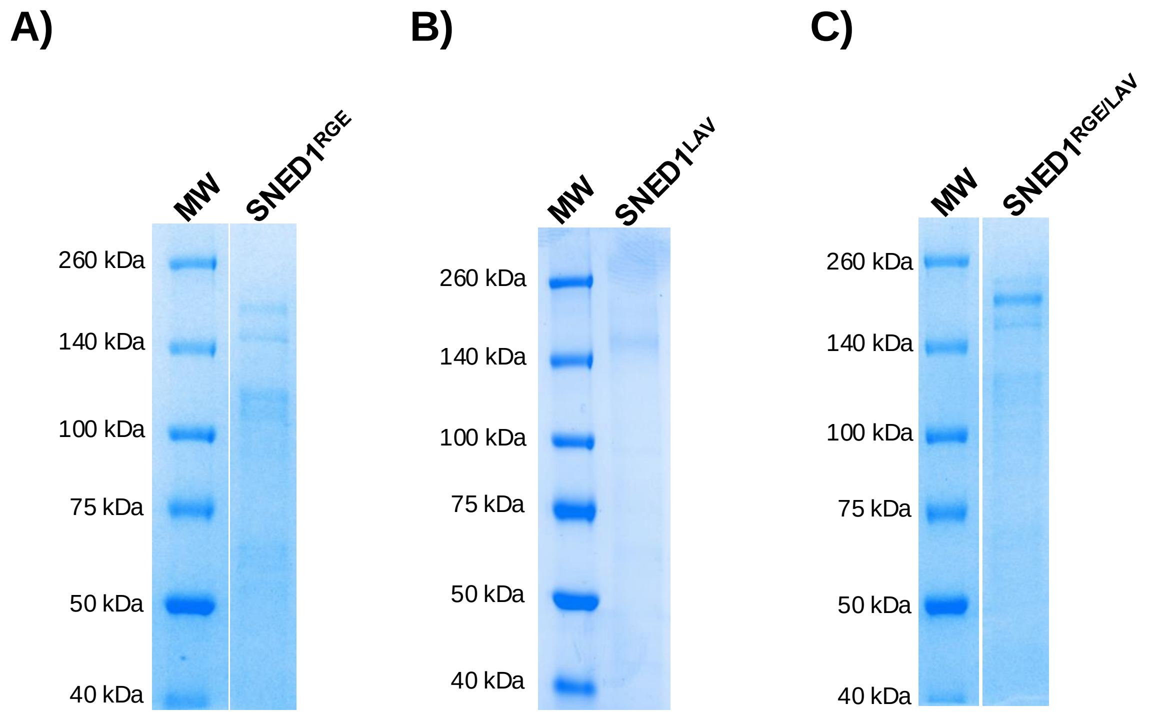

Fig. S2. Purification of integrin binding mutants of SNED1

Coomassie-blue-stained polyacrylamide gels showing the quality and purity of affinity-purified His-tagged SNED1^{RGE} (**A**), SNED1^{LAV} (**B**), and SNED1$^{RGE/LAV}$ (**C**). The different bands correspond to different levels of glycosylation of the proteins, as previously shown (Vallet *et al.*, 2021).

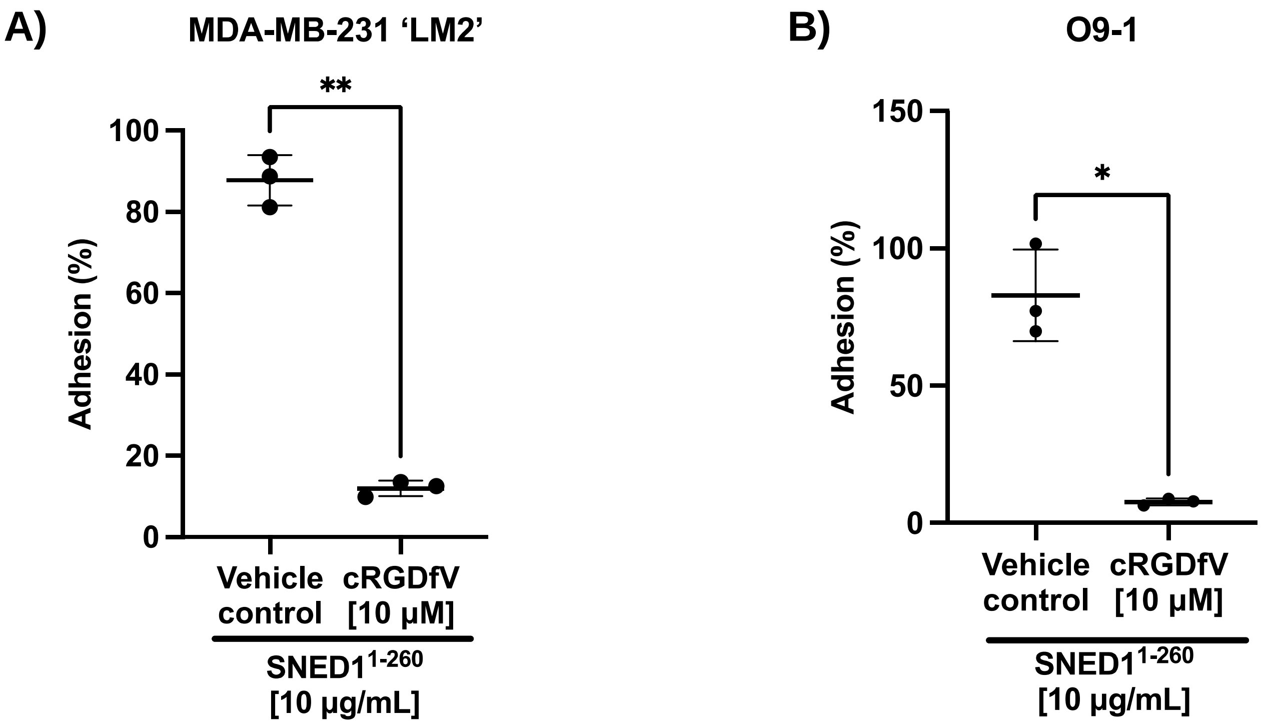

Fig. S3. The RGD motif in SNED1$^{1\text{-}260}$ is required for cell adhesion

Adhesion of MDA-MB-231' LM2' breast cancer cells (**A**) and O9-1 neural crest cells (**B**) to the N-terminal fragment of SNED1 (SNED1$^{1\text{-}260}$) is significantly decreased in presence of the integrin-binding cRGDfV peptide. Data is represented as mean ± SD from three biological replicates. Unpaired Student's two-tailed t-test with Welch's correction was performed to determine statistical significance. *$P<0.05$, **$P<0.01$.

Journal of Cell Science • Supplementary information

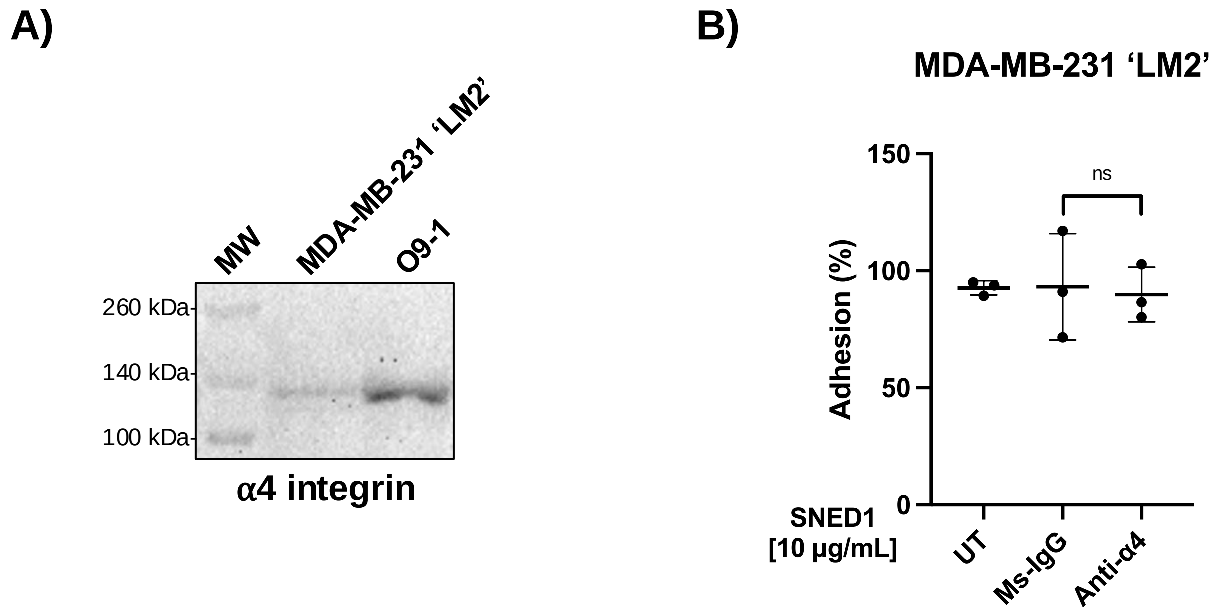

Fig. S4. Functional blocking of α4 integrin does not affect breast cancer cell adhesion to SNED1

A) Immunoblot on total cell extract from MDA-MB-231 'LM2' and O9-1 cells showing α4 integrin expression.
B) Adhesion of MDA-MB-231 'LM2' breast cancer cells to SNED1 is not altered in presence of anti-α4 integrin-blocking antibody. Data is represented as mean ± SD from three biological experiments. Unpaired Student's two-tailed t-test with Welch's correction was performed to test statistical significance. ns: non-significant.

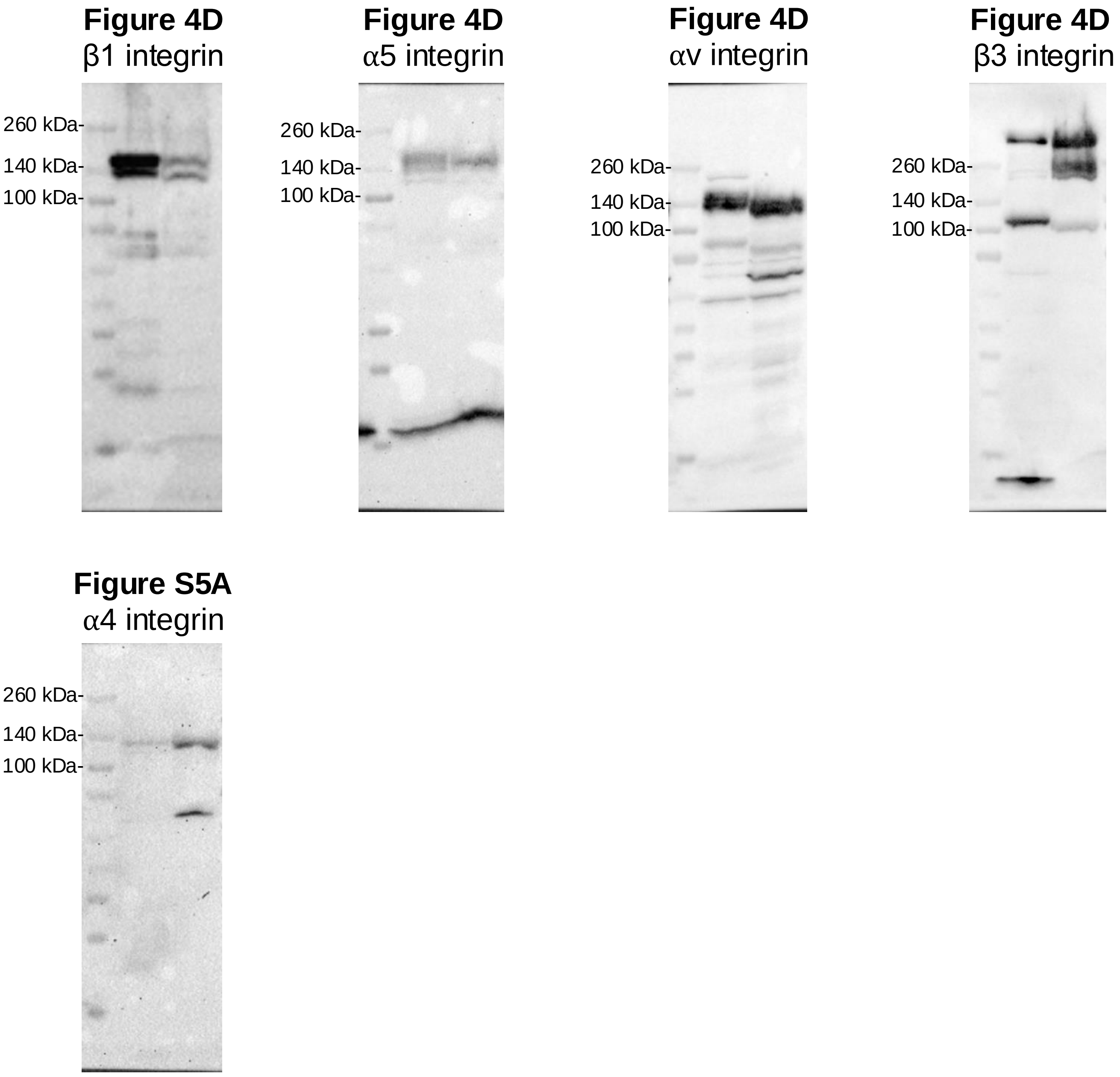

Fig. S5. Immunoblot transparency

Uncropped immunoblots for β1 integrin, α5 integrin, αv integrin, β3 integrin and α4 integrin.

Journal of Cell Science • Supplementary information

Table S1. List of primers

Primer	Sequence	Purpose
BglII-KOZAK-ATG-SNED1Hs_F	5’ TCGAAGATCTGCCACCATGCGGCACGGCGTCGC 3’	Subcloning SNED1 into retroviral pMSCV-IRES-Hygro vector
HpaI-Stop-His-SNED1Hs_R	5’GATGTTAACTTAGTGGTGATGGTGATGATGAGATTTCTCCAGTGTCTGACTCTTACT 3’	Shuttling of SNED1-His WT, RGE, LAV, or GE/LAV) into retroviral pMSCV-IRES-Hygro vector
HpaI-Stop-FLAG-NFS_R	5’ CCGTTAACTTACTTGTCGTCATCGTCTTTGTAGTCGCACTGGGGAGGCTCAC 3’	Subcloning SNED1$^{1-571}$ into retroviral pMSCV-IRES-Hygro vector with the addition of a C-terminal FLAG tag
HpaI-Stop-FLAG-NF_R	5’ CCGTTAACTTACTTGTCGTCATCGTCTTTGTAGTCGACGCAGAGGTAGCTCCC 3’	Subcloning SNED1$^{1-530}$ into retroviral pMSCV-IRES-Hygro vector with the addition of a C-terminal FLAG tag
SNED1_c120a _F	5’ CCGAGCGCGGCGAAGCCGTCACC 3’	Introduction of the c120>a (pG40E) point mutation to generate SNED1^{RGE}
SNED1_c120a _R	5’ GGTGACGGCTTCGCCGCGCTCGG 3’	
SNED1_a932c_F	5’ GGAGGTGCCACCTGGCCGTGAACGAATGTGC 3’	Introduction of the a932>c (pD311A) point mutation to generate SNED1^{LAV}
SNED1_a932c_R	5’ GCACATTCGTTCACGGCCAGGTGGCACCTCC 3’	

Journal of Cell Science • Supplementary information

Table S2. List of antibodies used for functional blocking and immunoblotting

Antibody	Host species	Reactivity	Application	Concentration used (µg/mL)	Catalog #
Anti-β1 Integrin	Rat	Human	Functional blocking	10	Sigma, MABT821
Anti-α5 Integrin	Rat	Human	Functional blocking	10	Sigma, MABT820
Anti-α4 Integrin	Mouse	Human	Functional blocking	10	Sigma, MAB1383
Anti-β1 Integrin	Hamster	Mouse	Functional blocking	10	BD Biosciences, BDB555002
Anti-α5 Integrin	Rat	Mouse	Functional blocking	10	Biolegend, 103817
Anti-αvβ3 Integrin	Mouse	Human	Functional blocking	10	Sigma, MAB1976Z
Rat IgG	Rat	Isotype control	Functional blocking	10	Invitrogen, PI31903
Mouse IgG	Mouse	Isotype control	Functional blocking	10	Invitrogen, PI31933
Hamster IgM	Hamster	Isotype control	Functional blocking	10	Biolegend, 401014
Anti-SNED1	Rabbit	Human	Immunoblotting	1	Naba lab
Anti-His	Mouse	-	Immunoblotting	1	Sigma, SAB2702218
Anti-FLAG	Rabbit	-	Immunoblotting	1	Sigma, F7425
Anti-β1 Integrin sera	Rabbit	Human, Mouse	Immunoblotting	1:1000 dilution	In-house
Anti-α5 Integrin	Rabbit	Human, Mouse	Immunoblotting	0.217	Abcam, AB150361
Anti-α4 Integrin	Rabbit	Human, Mouse	Immunoblotting	0.5	Invitrogen, MA5-27947
Anti-αv Integrin	Rabbit	Human, Mouse	Immunoblotting	1	Invitrogen, MA5-32195
Anti-β3 Integrin	Rabbit	Human, Mouse	Immunoblotting	1	Invitrogen, MA5-32077

Journal of Cell Science • Supplementary information
