# Supplementary material for: The novel ECM protein SNED1 mediates cell adhesion via the RGD-binding integrins α5β1 and αvβ3
Source: J Cell Sci. 2025 Jan 22;138(2):JCS263479. doi: 10.1242/jcs.263479 (PMC11828466; doi:10.1242/jcs.263479)
Supplement: Supplementary file 1 [file joces-138-263479_review_history.pdf]

Journal of Cell Science

The novel ECM protein SNED1 mediates cell adhesion via the RGD-binding integrins $\alpha5\beta1$ and $\alpha v\beta3$

Dharma Pally, Nandini Kapoor and Alexandra Naba
DOI: 10.1242/263479

Editor: Kathleen Green

Review timeline
Original submission: 8 August 2024
Editorial decision: 29 August 2024
First revision received: 25 November 2024
Accepted: 15 December 2024

Original submission

First decision letter

MS ID#: jcs.263479

MS TITLE: The novel ECM protein SNED1 mediates cell adhesion via α5ß1 integrin

AUTHORS: Dharma Pally; Nandini Kapoor; Alexandra Naba

ARTICLE TYPE: Short Report

Dear Dr Naba,

We have now reached a decision on the above manuscript.

To see the reviewers' reports and a copy of this decision letter, please go to:

As you will see, the reviewers raise a number of substantial criticisms that prevent me from accepting the paper at this stage. They suggest, however, that a revised version might prove acceptable, if you can address their concerns. If you think that you can deal satisfactorily with the criticisms on revision, I would be pleased to see a revised manuscript. We would then return it to the reviewers.

Reviewer 1

Advance summary and potential significance to field

This short report reports the identification of the alpha5-beta1 integrin as mediating cell adhesion by two biomedically interesting cell lines to the ECM protein SNED1. This ECM protein contains both RGD and LDV sites, but mutational analysis in this paper indicates that only the RGD site is involved in the in vitro adhesion assay used by the authors. A cyclic RGD inhibitor and anti-functional antibodies support the importance of the RGD sequence, with antibody producing about 50% inhibition of adhesion of one cell line to SNED1 (but quite minimal inhibition for the second line). In general, this manuscript is well-written and presented carefully. showing all data points for the biological replicates. The overall conclusion about the receptor for SNED1 will be useful and significant for the field if it can be demonstrated convincingly. The conclusion about the alpha5-

© 2025. Published by The Company of Biologists under the terms of the Creative Commons Attribution License (https://creativecommons.org/licenses/by/4.0/).

beta1 integrin being the receptor mediating adhesion to SNED1 is plausible with the significant reservations indicated below that will need addressing.

Comments for the author

1. The authors state in the Abstract: "cell adhesion to SNED1 is mediated by α5β1integrin." However, the RGD integrin receptor by far the most sensitive to cyclic RGDfV is alphaV-beta3. Unless the authors have evidence for a sequence in SNED1 that could function as the "synergy" sequence used by alpha5-beta1 (a5b1) and most importantly can rule out alphaV integrins, the evidence for a5b1 as the receptor mediating adhesion to SNED1 is currently too weak. It is not clear why the authors have not tested for the role of the most obvious receptor candidate aVb3, especially considering that the O9-1 cells were barely inhibited in adhesion to SNED1 by the anti-a5 antibody. The authors need to test a good anti-functional aV antibody for inhibitory activity. Although one could ideally ask for various control experiments to validate activities of all the antibodies (but not essential), a control to confirm aVb3 antibody activity, if there is no inhibition, could be to show effects of the anti-aV antibody on cell adhesion to fibronectin.

2. The SNED1 truncational mutagenesis studies are useful, with the interesting finding that partial truncation is less functionally active than major truncation. Although the authors' suggestion that this finding could indicate some conformational feature, a key point is that the assays were all performed at 10 ug/mL of all constructs. The molar concentration of the 1-260 construct would therefore be 6-7 times greater than the full-length molecule, so the statement that "it mediated cell adhesion at the same extent as SNED1…." Is not correct for equimolar concentrations. If they still have this truncated protein available, they should test equimolar concentrations, since the other possibility is that activity will fall for all constructs after truncation. However, if this will be too difficult to test, at the minimum the authors need to indicate this major caveat.

3. The authors need to indicate which antibodies were used for the inhibitory data shown because they list both two anti-a5 and two anti-b1 antibodies (one of the latter says it is anti-mouse with no confirmation about human on the company website).

4. A puzzle: Although the protein staining shown in Figure S2,B does not seem to show any protein of the expected size, the fact that it seems quite active may make this concern unimportant but nevertheless puzzling. This is not a major concern considering that there was no antibody inhibition of a4.

5. Very minor point: The authors indicate that samples were "boiled" at 95C, but boiling occurs at 100C. They could just state that the samples were heated to 95C.

Reviewer 2

Advance summary and potential significance to field

Previous studies from this group identified a role for the extracellular matrix-associated protein, SNED1, as a promoter of breast cancer metastasis and as essential in neural crest cells for craniofacial development. However, SNED1 is relatively underexplored among matrix proteins, and the mechanisms through which it exerts its effects have been unknown. In this study the authors use pharmacological and molecular genetic approaches to demonstrate that SNED1 mediates adhesion of breast cancer cells (MDA-MB-231 'LM2') or neural crest cells (O9-1) via an RGD motif within its N-terminal domain, and they identify the integrin α5β1 as a receptor in these cells that mediates adhesion to SNED1. This is a straightforward study of adhesion receptor identification; the novel findings are solid and contribute to our understanding of how SNED1 may exert its functional effects on cells and cause the in vivo phenotypes that have been observed previously in SNED1-deficient mice/tissues. The study would be improved substantially by inclusion of some cell functional assays (e.g., fiber assembly, see specific comment 3 below), to strengthen the link of the adhesion findings reported here to the previously observed physiological functions of SNED1.

Comments for the author

© 2025. Published by The Company of Biologists under the terms of the Creative Commons Attribution License (https://creativecommons.org/licenses/by/4.0/).

Specific comments:

1. Data in Fig. 4F show that blocking alpha5 integrin has only a modest effect on O9-1 cell adhesion to SNED1, while the effect of blocking beta1 integrins is substantial (which the authors acknowledge). This finding suggests involvement of another RGD-binding beta1 integrin. Have the authors tested blocking different RGD-binding integrins in combination to test for compensation?

2. Also regarding Fig. 4F, have the authors tested whether blocking alpha5beta1 abolishes O9-1 cell adhesion to the 1-260 SNED1 fragment? Perhaps the greater effect of blocking beta1 occurs through other SNED1 domains.

3. Page 8, lines 58-61: The authors suggest the interesting possibility that alpha5beta1 mediates SNED1 fiber assembly. These experiments are not outside the scope of the current study and should be included; in fact, they seem especially important given classic studies from the Hynes group and others who showed distinct and cooperative roles of the RGD motif and other domains within FN in cell adhesion and fiber assembly. Such experiments should seem very easy to carry out as this group has all reagents in hand and has established SNED1 fiber assembly assays (Vallet et al., 2021).

Minor comments:

4. It would be useful to mention (or illustrate) the evolutionary relatedness of human and murine SNED1, given that the authors compare them functionally.

5. Page 6, lines 4-6: The authors state "These synthetic constructs were designed to include adequate domain boundaries to ensure proper folding...(Fig. 2B)." "Ensure" seems too strong a word here. In fact, later in the same paragraph it is suggested that differences in adhesion to different truncations could be due to a requirement for FN3 and/or EGF-like domains for full-length SNED1 to adopt a confirmation where the N-terminal adhesive site is most accessible, suggesting that folding/confirmation was affected in some of the truncations.

6. Page 7, lines 34-38: "This result suggests that the adhesive property of SNED1(1-260) is solely mediated by the RGD motif and not the NIDO domain." I suggest modifying this conclusion a bit as it suggests that other receptors are not involved at all in cell adhesion to SNED1, which remains an open question (as the authors discuss later). The data show that the RGD is essential, but they don't rule out a role for co-receptors that may bind to other sites in the NIDO domain.

7. Which of the potential integrin receptors for SNED1 (shown in Fig. 4C) are known to be expressed by MDA-MB-231 cells or O9-1 cells?

8. In Figure 1, it's not clear whether "100% adhesion" represents 100% of the cells plated, or the maximum level of adhesion based on the standards that they used.

9. Page 6, lines 17-19: correct "SNED1(1-750)" to "SNED1(1-751)".

10. The title over Figure S4 should be corrected to state that blocking of alpha4 integrin does NOT affect breast cancer cell adhesion (the figure legend title is correct).

First revision

<u>Author response to reviewers' comments</u>

We thank the reviewers for their enthusiastic assessment of our manuscript and for providing valuable feedback. We were pleased to read that the reviewers found our manuscript “well-written and presented carefully” and for recognizing our findings as “novel”, “solid”, and of “significance for the field”. We are providing here a point-by-point response to the reviewers’ comments and have revised the manuscript accordingly.

© 2025. Published by The Company of Biologists under the terms of the Creative Commons Attribution License (https://creativecommons.org/licenses/by/4.0/).

Reviewer 1:

SUMMARY OF THE ADVANCE MADE IN THIS PAPER AND ITS POTENTIAL SIGNIFICANCE TO THE FIELD
This short report reports the identification of the alpha5-beta1 integrin as mediating cell adhesion by two biomedically interesting cell lines to the ECM protein SNED1. This ECM protein contains both RGD and LDV sites, but mutational analysis in this paper indicates that only the RGD site is involved in the in vitro adhesion assay used by the authors. A cyclic RGD inhibitor and anti- functional antibodies support the importance of the RGD sequence, with antibody producing about 50% inhibition of adhesion of one cell line to SNED1 (but quite minimal inhibition for the second line). In general, this manuscript is well-written and presented carefully. showing all data points for the biological replicates. The overall conclusion about the receptor for SNED1 will be useful and significant for the field if it can be demonstrated convincingly. The conclusion about the alpha5-beta1 integrin being the receptor mediating adhesion to SNED1 is plausible with the significant reservations indicated below that will need addressing.

SUGGESTIONS TO AUTHORS
1. The authors state in the Abstract: "cell adhesion to SNED1 is mediated by α5β1integrin." However, the RGD integrin receptor by far the most sensitive to cyclic RGDfV is alphaV-beta3. Unless the authors have evidence for a sequence in SNED1 that could function as the "synergy" sequence used by alpha5-beta1 (a5b1) and most importantly can rule out alphaV integrins, the evidence for a5b1 as the receptor mediating adhesion to SNED1 is currently too weak. It is not clear why the authors have not tested for the role of the most obvious receptor candidate aVb3, especially considering that the O9-1 cells were barely inhibited in adhesion to SNED1 by the anti- a5 antibody. The authors need to test a good anti-functional aV antibody for inhibitory activity. Although one could ideally ask for various control experiments to validate activities of all the antibodies (but not essential), a control to confirm aVb3 antibody activity, if there is no inhibition, could be to show effects of the anti-aV antibody on cell adhesion to fibronectin.

> We thank the reviewer for this suggestion. We have now confirmed the expression of integrins αv and β3 expression in both LM2 and O9-1 cells using immunoblot, see **revised Figure 4D**.
We have also performed cell adhesion assay in presence of functional blocking antibody targeting the αvβ3 integrin and have observed a significant decrease (35.5% decrease) in LM2 cell adhesion to SNED1 in presence of that antibody when compared to isotype control. These new findings have been added to **revised Figure 4E** described page 8, lines 228-233 of the revised manuscript. We were not able to test the effect of blocking αvβ3 integrin on O9-1 cells as we could not find a suitable functional blocking antibody targeting the murine form of αvβ3 integrin. However, we hope to be able to perform similar experiment in the future as we pursue our investigations of the roles of SNED1/integrin interaction on cell phenotype.

2. The SNED1 truncational mutagenesis studies are useful, with the interesting finding that partial truncation is less functionally active than major truncation. Although the authors' suggestion that this finding could indicate some conformational feature, a key point is that the assays were all performed at 10 ug/mL of all constructs. The molar concentration of the 1-260 construct would therefore be 6-7 times greater than the full-length molecule, so the statement that "it mediated cell adhesion at the same extent as SNED1…." Is not correct for equimolar concentrations. If they still have this truncated protein available, they should test equimolar concentrations, since the other possibility is that activity will fall for all constructs after truncation. However, if this will be too difficult to test, at the minimum the authors need to indicate this major caveat

> We thank the reviewer for this comment. At a concentration of 10 μg/mL $SNED1^{1-260}$ is indeed at a concentration of 375.94 μM while the full length is at a concentration of 66.3 μM. To address the reviewer's comment, we have repeated the assay on substrate coated with 66.3 μM of full length SNED1 or $SNED1^{1-260}$ and have observed that at a concentration of 66.3 μM $SNED1^{1-260}$ still mediates the adhesion of LM2 cells, although we noted a small decrease as compared to lower compared to full length SNED1 (69% vs 81%, respectively). These results are presented in the new **Figure S2** and described page 6, lines 148-151 of the revised manuscript. Importantly, cell adhesion on 66.3 μM (1.34 μg/mL) $SNED1^{1-260}$ is still higher compared to $SNED1^{1-751}$ and $SNED1^{1-530}$ at 10 μg/mL suggesting that the shortest truncated form is sufficient to mediate cell

© 2025. Published by The Company of Biologists under the terms of the Creative Commons Attribution License (https://creativecommons.org/licenses/by/4.0/).

adhesion to a comparable proportion as to full length SNED1 as we had reported in the original version of the manuscript.

3. The authors need to indicate which antibodies were used for the inhibitory data shown because they list both two anti-a5 and two anti-b1 antibodies (one of the latter says it is anti-mouse with no confirmation about human on the company website).

> We apologize for the confusion. LM2 cells are of human origin and O9-1 cells are mouse neural crest cells. Hence, the antibodies with catalog numbers MABT821 and MABT820 have specificity towards human β1 and α5 integrins only. The antibodies with catalog numbers BDB555002 and 103817, based on the existing literature, have reactivity against mouse β1 and α5 integrins only.

4. A puzzle: Although the protein staining shown in Figure S2,B does not seem to show any protein of the expected size, the fact that it seems quite active may make this concern unimportant but nevertheless puzzling. This is not a major concern considering that there was no antibody inhibition of a4.

> The expected molecular weight of SNED1 is approximately 154 kDa. We previously conducted the biochemical characterization of SNED1 and have reported that it exists in multiple glycoforms (Vallet *et al.*, Biochemical J, 2021). The protein staining observed in **Figure S3B** of the revised manuscript (originally Figure S2B) corresponds to one of the glycoforms of SNED1.

5. Very minor point: The authors indicate that samples were "boiled" at 95C, but boiling occurs at 100C. They could just state that the samples were heated to 95C.

> We thank the reviewer for bringing this our attention. We have now replaced "boiled" by "heated" in the revised manuscript, see page 14, line 436 of the revised manuscript.

Reviewer **2:**

SUMMARY OF THE ADVANCE MADE IN THIS PAPER AND ITS POTENTIAL SIGNIFICANCE TO THE FIELD
Previous studies from this group identified a role for the extracellular matrix-associated protein, SNED1, as a promoter of breast cancer metastasis and as essential in neural crest cells for craniofacial development. However, SNED1 is relatively underexplored among matrix proteins, and the mechanisms through which it exerts its effects have been unknown. In this study the authors use pharmacological and molecular genetic approaches to demonstrate that SNED1 mediates adhesion of breast cancer cells (MDA-MB-231 'LM2') or neural crest cells (O9-1) via an RGD motif within its N-terminal domain, and they identify the integrin α5β1 as a receptor in these cells that mediates adhesion to SNED1. This is a straightforward study of adhesion receptor identification; the novel findings are solid and contribute to our understanding of how SNED1 may exert its functional effects on cells and cause the in vivo phenotypes that have been observed previously in SNED1-deficient mice/tissues. The study would be improved substantially by inclusion of some cell functional assays (e.g., fiber assembly, see specific comment 3 below), to strengthen the link of the adhesion findings reported here to the previously observed physiological functions of SNED1.

SUGGESTIONS TO AUTHORS

Specific comments:
1. Data in Fig. 4F show that blocking alpha5 integrin has only a modest effect on O9-1 cell adhesion to SNED1, while the effect of blocking beta1 integrins is substantial (which the authors acknowledge). This finding suggests involvement of another RGD-binding beta1 integrin. Have the authors tested blocking different RGD-binding integrins in combination to test for compensation?

> Thank you for this comment. Indeed, our result subject that another β1-containing dimer may serve as a SNED1 receptor. There are 3 RGD-binding integrin heterodimers that contain β1: α5β1, αvβ1 (primarily expressed by activated fibroblasts), and α8β1 and there are additional β1-containing dimers that are RGD-independent. Of note, only α5β1 was predicted to interact with SNED1 (see molecular modeling data published in Vallet *et al.*, Biochemical J, 2021). As such, as part of this short report, we have not tested the effect of blocking other partners of β1 in

© 2025. Published by The Company of Biologists under the terms of the Creative Commons Attribution License (https://creativecommons.org/licenses/by/4.0/).

combination to test for compensation. Since we are now showing that αv integrin is expressed by both LM2 and 09-1 cells (see **new Figure 4D**), we can speculate that αvβ1 is another potential receptor of SNED1 (we now discuss this page 8, line 238 of the revised manuscript). However were not able to perform this experiment in the time imparted. We were also not able to test the relevance of α8 integrin due to lack of functional-blocking reagents, but it is something we can envision doing in the future.

2. Also regarding Fig. 4F, have the authors tested whether blocking alpha5beta1 abolishes 09-1 cell adhesion to the 1-260 SNED1 fragment? Perhaps the greater effect of blocking beta1 occurs through other SNED1 domains.

> We have not tested the ability of 09-1 cell to adhere on $SNED1^{1-260}$ in presence of functional blocking antibodies against α5 or β1 integrin. However, we have observed a drastic decrease in 09-1 cell adhesion to $SNED1^{1-260}$ upon inhibiting RGD-binding integrins using cRGDfV peptide (see Figure S4B). We thus can reasonably speculate that blocking α5 and β1 integrins will also result in a similar decrease.

3. Page 8, lines 58-61: The authors suggest the interesting possibility that alpha5beta1 mediates SNED1 fiber assembly. These experiments are not outside the scope of the current study and should be included; in fact, they seem especially important given classic studies from the Hynes group and others who showed distinct and cooperative roles of the RGD motif and other domains within FN in cell adhesion and fiber assembly. Such experiments should seem very easy to carry out as this group has all reagents in hand and has established SNED1 fiber assembly assays (Vallet et al., 2021).
> We acknowledge the reviewer's valuable suggestion but feel that this falls beyond the scope of the current *Short Report* manuscript. A graduate student in our laboratory is actively investigating the mechanisms of SNED1 assembly in the ECM and part of her project is to determine whether these mechanisms involve integrins.

Minor comments:

4. It would be useful to mention (or illustrate) the evolutionary relatedness of human and murine SNED1, given that the authors compare them functionally.

> Thank you for this comment. We have previously reported the evolutionary relatedness of human and murine SNED1 (84% identity) in a previous publication (Barqué et al., 2021). We have now included a statement mentioning this, see page 5, lines 118-119 of the revised manuscript.

5. Page 6, lines 4-6: The authors state "These synthetic constructs were designed to include adequate domain boundaries to ensure proper folding…(Fig. 2B)." "Ensure" seems too strong a word here. In fact, later in the same paragraph it is suggested that differences in adhesion to different truncations could be due to a requirement for FN3 and/or EGF-like domains for full- length SNED1 to adopt a confirmation where the N-terminal adhesive site is most accessible, suggesting that folding/confirmation was affected in some of the truncations.

> We apologize for the confusion. In the sentence "These synthetic constructs were designed to include adequate domain boundaries to ensure proper folding…" we did not mean to comment on proper *conformation* to mediate functions, we meant to refer to the adoption of a proper *folding* required for secretion into extracellular space, as opposed to misfolded proteins that would undergo intracellular degradation. We have now revised the wording, see page 6, lines 138-139.

6. Page 7, lines 34-38: "This result suggests that the adhesive property of SNED1(1-260) is solely mediated by the RGD motif and not the NIDO domain." I suggest modifying this conclusion a bit as it suggests that other receptors are not involved at all in cell adhesion to SNED1, which remains an open question (as the authors discuss later). The data show that the RGD is essential, but they don't rule out a role for co-receptors that may bind to other sites in the NIDO domain.

> We absolutely agree. We have now modified this statement, see page 7, lines 201-203 of the revised manuscript.

© 2025. Published by The Company of Biologists under the terms of the Creative Commons Attribution License (https://creativecommons.org/licenses/by/4.0/).

7. Which of the potential integrin receptors for SNED1 (shown in Fig. 4C) are known to be expressed by MDA-MB-231 cells or O9-1 cells?

> There is limited published information on the panel of integrins expressed by these cell lines. One study has shown that LM2 cells express integrin α5β1 and αVβ1 (Shen et al., 2019), which is in line with our own results (Figure 4D). We also have unpublished RNA-seq data from LM2 tumor xenografts showing that these cells, *in vivo*, express the following integrins: *ITGA1*, *ITGA2*, *ITGA3*, *ITGA5*, *ITGA6*, *ITGA10*, *ITGAV*, *ITGB1*, *ITGB5*, *ITGB4*. This data is only for the reviewer's information and not included in the revised manuscript.
The O9-1 cell line is the only commercially available neural crest cell line and, to the best of our knowledge, there are no published studies reporting the integrin expression profile of these cells.

8. In Figure 1, it's not clear whether "100% adhesion" represents 100% of the cells plated, or the maximum level of adhesion based on the standards that they used.

> We apologize for the confusion. The 100% adhesion in Figure 1 represents the maximum level of adhesion relative to the standard used in the experiment. We have now added this to the text, see page 5, line 116 of the revised manuscript

9. Page 6, lines 17-19: correct "SNED1(1-750)" to "SNED1(1-751)".

> We thank the reviewer for pointing out this inadvertent mistake. We have corrected this in the revised manuscript (see page 6, lines 146 and 147).

10. The title over Figure S4 should be corrected to state that blocking of alpha4 integrin does NOT affect breast cancer cell adhesion (the figure legend title is correct).

> We sincerely thank the reviewer for bringing this to our attention. We have now corrected this to "Functional blocking of α4 integrin does not affect breast cancer cell adhesion to SNED1" in the revised manuscript.

References:

Barqué, A., Jan, K., De La Fuente, E., Nicholas, C. L., Hynes, R. O. and Naba, A. (2021). Knockout of the gene encoding the extracellular matrix protein SNED1 results in early neonatal lethality and craniofacial malformations. *Developmental Dynamics* **250**, 274–294.

Shen, M., Jiang, Y.-Z., Wei, Y., Ell, B., Sheng, X., Esposito, M., Kang, J., Hang, X., Zheng, H., Rowicki, M., et al. (2019). Tinagl1 Suppresses Triple-Negative Breast Cancer Progression and Metastasis by Simultaneously Inhibiting Integrin/FAK and EGFR Signaling. *Cancer Cell* **35**, 64-80.e7.

Vallet, S. D., Davis, M. N., Barqué, A., Thahab, A. H., Ricard-Blum, S. and Naba, A. (2021). Computational and experimental characterization of the novel ECM glycoprotein SNED1 and prediction of its interactome. *Biochemical Journal* **478**, 1413-1434.

Second decision letter

MS ID#: jcs.263479R1

MS Title: The novel ECM protein SNED1 mediates cell adhesion via the RGD-binding integrins α5β1 and αvβ3

Authors: Dharma Pally; Nandini Kapoor; Alexandra Naba

Article Type: Short Report

© 2025. Published by The Company of Biologists under the terms of the Creative Commons Attribution License (https://creativecommons.org/licenses/by/4.0/).

Dear Dr Naba,

I am happy to tell you that your manuscript has been accepted for publication in Journal of Cell Science, pending standard publication integrity checks. It was accepted on 15 Dec 2024. Where referee reports on this version are available, they are appended below.

Reviewer 1

Advance summary and potential significance to field

See previous review for summary. The authors have responded directly, comprehensively, and sufficiently to all my concerns and suggestions. The addition of a second integrin receptor is very helpful and makes sense based on the initial data.

I feel that they have responded adequately, within reason, to the concerns of the other reviewer - I would oppose asking them to extend this short report any further.

Overall, this paper makes a significant contribution to the field and now seems ready for publication in JCS.

Comments for the author

None - acceptance is recommended.

Reviewer 2

In this revised manuscript, the authors have addressed previous concerns. It is unfortunate that experiments to assess roles for these integrins in mediating SNED1 fiber assembly are considered outside the scope of this study, as they would enhance the impact of these findings. However, it is understood that this is a Short Report, and the addition of new experiments to consider contributions by integrin alphaV beta3 do strengthen the study.

© 2025. Published by The Company of Biologists under the terms of the Creative Commons Attribution License (https://creativecommons.org/licenses/by/4.0/).
